# A new model for educational programming in global health emerges during COVID-19

**DOI:** 10.7189/jogh.11.03034

**Published:** 2021-02-11

**Authors:** Chao Long, Natalie Meyers, Tingadini Nyoni, Dharshan Sivaraj, Godfrey I Muguti, James Chang

**Affiliations:** 1 Department of Plastic and Reconstructive Surgery, Johns Hopkins Hospital, Baltimore, Maryland, USA; 2 ReSurge International, Mountain View, California, USA; 3 The College of Surgeons of East, Central, and Southern Africa (COSECSA), Arusha, Tanzania; 4 Stanford University School of Medicine, Stanford, California, USA; 5 Department of Surgery, University of Zimbabwe, Harare, Zimbabwe; 6 Division of Plastic and Reconstructive Surgery, Department of Surgery, Stanford University Medical Center, Stanford, California, USA

COVID-19 has disrupted in-person medical training worldwide. Although there has been a discussion of innovative educational solutions for high-income country (HIC) trainees [1-5], it is lacking for trainees in low- and middle-income countries (LMICs). This is problematic because there is already a shortage of trained medical professionals in LMICs [6]. Additionally, this shortage is felt most acutely in surgery and anesthesia. While one third of the world’s population lives in Africa and southeast Asia, only 12% of the world’s specialist surgeons practice there [7]. In this article, we discuss the impact of COVID-19 on medical and surgical educational programming for trainees in LMICs, and use a case study to provide recommendations.

Many international non-governmental organizations (NGOs) and academic institutions in HICs have invested in building the global health care workforce through bidirectional partnerships and educational programming such as training workshops [8]. These in-person training visits, designed to supplement local training programs, came to a halt during COVID-19. Recognizing the importance of continuing to provide this education, virtual curricula have been developed and deployed via video conferencing applications.

This virtual format confers numerous advantages. Most notably, without the need to travel to LMICs, there are substantial cost and time savings for educators. Trainees can gain access to the most sought-after educators who previously could not participate due to time and financial restraints. This levels the playing field between LMIC and HIC trainees in terms of access to diverse content and curricula. A virtual platform can be accessed by more trainees and with greater ease, as trainees are also not required to travel to the instruction site. With higher attendance, the virtual format expands the programming’s reach, as it crosses hospitals, cities, countries, and continents. Ability and ease of recording this programming further increase its reach and utilization. These recordings form a repository of educational material, which trainees can access repeatedly as needed and which can be provided to future cohorts of trainees. Altogether, these advantages make sustainability – the ever-elusive goal for all forms of global health programming – more attainable.

**Figure:**
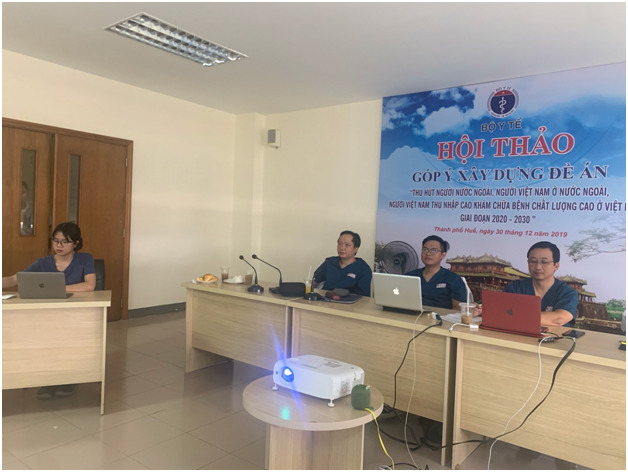
Photo: Colleagues in Vietnam participate in a virtual training session. (Source: ReSurge International, used with permission).

Virtual education however brings a unique set of challenges. First, trainees in LMICs may not have access to a reliable internet connection. In certain countries, there are frequent power outages that interfere with connectivity. Second, internet data, oftentimes paid out-of-pocket by trainees, may be prohibitively expensive given the high bandwidth and data usage that video streaming requires. Finally, because educators and trainees are typically in different time zones, the timing of the programming may preclude certain trainees from attending in real-time. Because of these challenges, we recommend that virtual educational programs consider providing financial or technical support to create a virtual learning environment locally. We also recommend all training to be recorded, so that these lessons can be watched asynchronously at any later time.

ReSurge International, an NGO dedicated to increasing access to reconstructive surgery, serves as an illustrative case example for this shift in educational programming. ReSurge provides surgical education and training via its Global Training Program [9,10]. Before COVID-19, this program comprised of Visiting Educators, a group of reconstructive surgeons who traveled to LMICs to deliver hands-on training. Once international travel was no longer possible, ReSurge quickly pivoted to a virtual curriculum involving several different lecture series, including a “Virtual Grand Rounds.” This curriculum was developed jointly with their LMIC partners. At the time of this article’s submission, it has reached more than 1200 participants from 24 countries spanning four continents. ReSurge implemented strategies to ensure useful, high quality content and close engagement from its trainees. For example, it conducts continuous needs assessments by surveying trainees and program directors. It also provides certificates of completion in order to incentivize attendance and participation. To measure its impact and track trainees’ progress, ReSurge administers pre- and post-lecture tests.

No global health educator would discount the value of being in-country and on-site; this allows us to perform needs assessments, build trust and goodwill with LMIC partners, understand the context of our work, and secure the buy-in that any successful global health initiative requires. Surgical education is furthermore unique from other types of medical education in that competence and mastery require not only knowledge and a command of surgical anatomy but also the technical skills for execution. For these reasons, we believe that post-COVID, educational programming in global health should reflect a hybrid curriculum including both virtual and in-person training. Before the visit, lectures can be delivered virtually, along with virtual pre-operative clinics to screen patients. This would be followed by in-person trainings that transfer diagnostic and technical skills to the trainees. Virtual training can continue after in-person trainings with additional lectures and virtual follow-up clinics. The proposed three-step hybrid program is likely to improve upon the primarily in-person models used pre-COVID because it more closely resembles existing surgical education paradigms that incorporate teaching of both technical skills and foundational knowledge. The addition of virtual training before and after in-person training allows for greater and continued emphasis on the knowledge component. This hybrid program can be repeated quarterly at host hospitals to create a continuous cycle of surgical education.

COVID-19 forced us to adapt, however it has also provided an opportunity to innovate and reimagine medical and surgical training. By leveraging modern technologies, virtual training and a hybrid curriculum offer a cheaper, less time-intensive, easily accessible, and more sustainable model for education and training in global health.
